# Supplementary material for: Anti-SARS-CoV-2 IgG Seroprevalence in Tyrol, Austria, among 28,768 Blood Donors between May 2022 and March 2023
Source: Vaccines (Basel). 2024 Mar 8;12(3):284. doi: 10.3390/vaccines12030284 (PMC10976183; doi:10.3390/vaccines12030284)
Supplement: Supplementary file 1 [file vaccines-12-00284-s001.zip › vaccines-2878803-supplementary.pdf]

Supplementary Materials

Anti-SARS-CoV-2 IgG Seroprevalence in Tyrol, Austria, among 28,768 Blood Donors between May 2022 and March 2023

Anita Siller et al.

Table of contents

Supplementary tables 3

Table S1. STROBE checklist. 3

Table S2. Differences in Spike RBD IgG antibody titre across groups defined by vaccination status and prior SARS-CoV-2 infection. 4

Table S3. Differences in Spike RBD IgG antibody titre by age and sex groups separated according the participants' vaccination status.. 5

Table S4. Differences in Spike RBD IgG antibody titre by age and sex groups separated according the participants' history of SARS-CoV-2 infection. 6

Supplementary figures 7

Figure S1. Age and sex distribution of individuals in the blood donor study population... 7

Figure S2. Percentage of participants in categories of Spike RBD IgG antibody titres in Binding Antibody Units per millilitre between May 2022 and March 2023. 8

Supplementary tables

Table S1. STROBE checklist.

	Item No	Recommendation	Page No
Title and abstract	1	(*a*) Indicate the study's design with a commonly used term in the title or the abstract	1
		(*b*) Provide in the abstract an informative and balanced summary of what was done and what was found	1
**Introduction**			
Background/ rationale	2	Explain the scientific background and rationale for the investigation being reported	1-2
Objectives	3	State specific objectives, including any prespecified hypotheses	2
**Methods**			
Study design	4	Present key elements of study design early in the paper	2
Setting	5	Describe the setting, locations, and relevant dates, including periods of recruitment, exposure, follow-up, and data collection	2
Participants	6	(*a*) Give the eligibility criteria, and the sources and methods of selection of participants. Describe methods of follow-up	2
		(*b*) For matched studies, give matching criteria and number of exposed and unexposed	NA
Variables	7	Clearly define all outcomes, exposures, predictors, potential confounders, and effect modifiers. Give diagnostic criteria, if applicable	2
Data sources/ measurement	8*	For each variable of interest, give sources of data and details of methods of assessment (measurement). Describe comparability of assessment methods if there is more than one group	2
Bias	9	Describe any efforts to address potential sources of bias	2-3
Study size	10	Explain how the study size was arrived at	2
Quantitative variables	11	Explain how quantitative variables were handled in the analyses. If applicable, describe which groupings were chosen and why	2-3
Statistical methods	12	(*a*) Describe all statistical methods, including those used to control for confounding	2-3
		(*b*) Describe any methods used to examine subgroups and interactions	2-3
		(*c*) Explain how missing data were addressed	2-3
		(*d*) If applicable, explain how loss to follow-up was addressed	NA
		(*e*) Describe any sensitivity analyses	2-3
**Results**			
Participants	13*	(a) Report numbers of individuals at each stage of study—eg numbers potentially eligible, examined for eligibility, confirmed eligible, included in the study, completing follow-up, and analysed	3-7
		(b) Give reasons for non-participation at each stage	3-7
		(c) Consider use of a flow diagram	NA
Descriptive data	14*	(a) Give characteristics of study participants (eg demographic, clinical, social) and information on exposures and potential confounders	3-4, Table 1
		(b) Indicate number of participants with missing data for each variable of interest	Table 1
		(c) Summarise follow-up time (eg, average and total amount)	3-7
Outcome data	15*	Report numbers of outcome events or summary measures over time	3-7
Main results	16	(a) Give unadjusted estimates and, if applicable, confounder-adjusted estimates and their precision (eg, 95% confidence interval). Make clear which confounders were adjusted for and why they were included	3-8
		(b) Report category boundaries when continuous variables were categorised	3-8
		(c) If relevant, consider translating estimates of relative risk into absolute risk for a meaningful time period	NA
Other analyses	17	Report other analyses done—eg analyses of subgroups and interactions, and sensitivity analyses	4-8
**Discussion**			
Key results	18	Summarise key results with reference to study objectives	9
Limitations	19	Discuss limitations of the study, taking into account sources of potential bias or imprecision. Discuss both direction and magnitude of any potential bias	11
Interpretation	20	Give a cautious overall interpretation of results considering objectives, limitations, multiplicity of analyses, results from similar studies, and other relevant evidence	9-11
Generalizability	21	Discuss the generalizability (external validity) of the study results	10-11
**Other information**			
Funding	22	Give the source of funding and the role of the funders for the present study and, if applicable, for the original study on which the present article is based	11

Table S2. Differences in Spike RBD IgG antibody titre across groups defined by vaccination status and prior SARS-CoV-2 infection.

	Geometric mean Spike RBD IgG (95% CI) in BAU/mL and *% differences compared the reference*		P interaction
	**By prior SARS-CoV-2 infection**		
	Yes[2]	No	
**By vaccination status**			
Vaccinated with booster[1]	2758 (2683 to 2835)	1150 (1072 to 1232)	0.216
	[Reference]	*[Reference]*	
Vaccinated without booster	1148 (1091 to 1208)	514 (400 to 662)	
	-58% (-61% to -55%)	*-55% (-63% to -45%)*	
Unvaccinated	144 (129 to 161)	31 (9 to 108)	
	-95% (-95% to -94%)	*-97% (-99% to -94%)*	

The analysis involved Spike RBD IgG antibody measurements taken from 8232 seropositive participants that completed the questionnaire in the period September 2022 to December 2022. To examine differences in Spike RBD IgG antibody titre according to prior SARS-CoV-2 vaccination and prior SARS-CoV-2 infection, we used a linear regression model adjusted for history of infection (no and yes), vaccination status (unvaccinated, vaccinated without booster, and vaccinated with booster), and the interaction between history of infection and vaccination status. [1]Refers to three or four SARS-CoV-2 vaccinations. [2]Refers to a SARS-CoV-2 infection prior to included Spike RBD IgG measurement detected by self-report or by seropositivity of Nucleocapsid IgG antibodies.

Table S3. Differences in Spike RBD IgG antibody titre by age and sex groups separated according the participants' vaccination status.

	Geometric mean Spike RBD IgG (95% CI) in BAU/mL and *% differences compared the reference*			P interaction
	**By vaccination status**			
	Yes, with booster[1]	Yes, without booster	Unvaccinated	
**By sex**				
Female	2132 (2044 to 2223)	1064 (984 to 1151)	134 (114 to 158)	0.593
	[Reference]	*[Reference]*	*[Reference]*	
Male	2250 (2162 to 2342)	1072 (998 to 1151)	150 (129 to 175)	
	6% (-0% to 12%)	*1% (-11% to 14%)*	*12% (-6% to 33%)*	
**By age group**				
18-24 years	2179 (2025 to 2344)	1198 (1063 to 1350)	113 (79 to 163)	0.283
	[Reference]	*[Reference]*	*[Reference]*	
25-34 years	2081 (1941 to 2232)	975 (877 to 1084)	130 (108 to 158)	
	-4% (-15% to 8%)	*-19% (-34% to -0%)*	*15% (-19% to 63%)*	
35-44 years	2041 (1910 to 2181)	962 (858 to 1080)	120 (95 to 150)	
	-6% (-17% to 5%)	*-20% (-35% to -1%)*	*5% (-25% to 49%)*	
45-54 years	2122 (2004 to 2247)	1051 (944 to 1170)	154 (125 to 189)	
	-3% (-12% to 8%)	*-12% (-28% to 7%)*	*35% (-4% to 90%)*	
55-64 years	2372 (2231 to 2523)	1198 (1026 to 1399)	176 (130 to 240)	
	9% (-2% to 21%)	*0% (-20% to 25%)*	*55% (9% to 121%)*	
65-70 years	2686 (2319 to 3111)	1708 (1246 to 2343)	273 (72 to 1031)	
	23% (5% to 44%)	*43% (-5% to 114%)*	*140% (24% to 364%)*	

The analysis involved Spike RBD IgG antibody measurements taken from 8232 seropositive participants that completed the questionnaire in the period September 2022 to December 2022. To examine differences in Spike RBD IgG antibody titre by age and sex group separated according participants' vaccination status, we used a linear regression model adjusted for sex (female and male) or age group (18 to <25, 25 to <35, 35 to <45, 45 to <55, 55 to <65, 65 to 70), vaccination status (unvaccinated, vaccinated without booster, and vaccinated with booster), and the interaction between sex or age group and vaccination status. [1]Refers to three or four SARS-CoV-2 vaccinations.

Table S4. Differences in Spike RBD IgG antibody titre by age and sex groups separated according the participants' history of SARS-CoV-2 infection.

	Geometric mean Spike RBD IgG (95% CI) in BAU/mL and *% differences compared the reference*		P interaction
	**By prior SARS-CoV-2 infection**		
	Yes[1]	No	
**By sex**			
Female	1607 (1530 to 1687)	1010 (908 to 1123)	0.947
	[Reference]	*[Reference]*	
Male	1773 (1694 to 1855)	1108 (1014 to 1212)	
	10% (3% to 18%)	*10% (-4% to 25%)*	
**By age group**			
18-24 years	1592 (1461 to 1734)	1360 (1090 to 1697)	<0.001
	[Reference]	*[Reference]*	
25-34 years	1364 (1260 to 1477)	968 (813 to 1152)	
	-14% (-24% to -3%)	*-29% (-47% to -4%)*	
35-44 years	1372 (1268 to 1485)	1004 (846 to 1192)	
	-14% (-24% to -2%)	*-26% (-45% to -1%)*	
45-54 years	1742 (1634 to 1858)	979 (861 to 1114)	
	9% (-3% to 23%)	*-28% (-45% to -6%)*	
55-64 years	2179 (2028 to 2341)	1114 (974 to 1273)	
	37% (21% to 54%)	*-18% (-37% to 7%)*	
65-70 years	3025 (2572 to 3558)	1331 (1006 to 1759)	
	90% (56% to 131%)	*-2% (-31% to 38%)*	

The analysis involved Spike RBD IgG antibody measurements taken from 8232 seropositive participants that completed the questionnaire in the period September 2022 to December 2022. To examine differences in Spike RBD IgG antibody titre by age and sex groups separated according participants' history of SARS-CoV-2 infection, we used a linear regression model adjusted for sex (female and male) or age group (18 to <25, 25 to <35, 35 to <45, 45 to <55, 55 to <65, 65 to 70), history of SARS-CoV-2 infection (yes, no), and the interaction between sex or age group and history of SARS-CoV-2 infection. [1]Refers to a SARS-CoV-2 infection prior to included Spike RBD IgG measurement detected by self-report or by seropositivity of Nucleocapsid IgG antibodies.

Supplementary figures

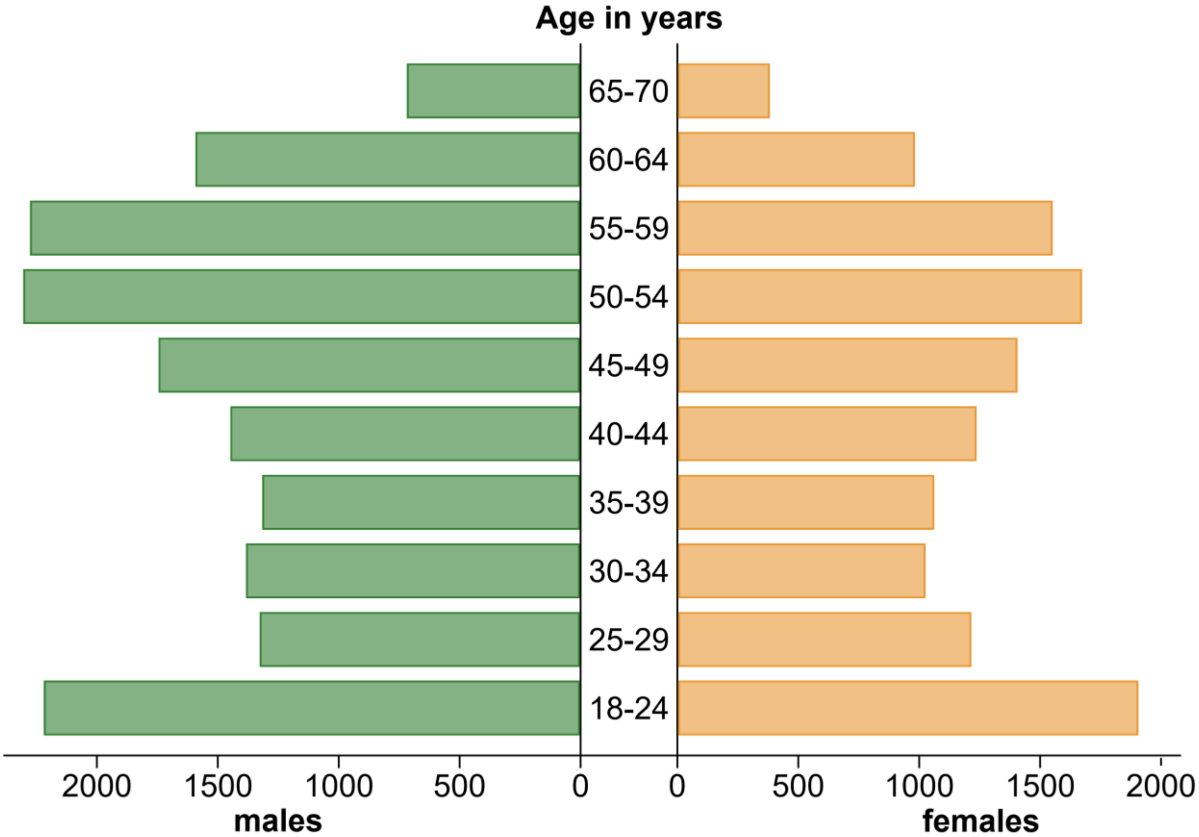

Figure S1. Age and sex distribution of individuals in the blood donor study population.

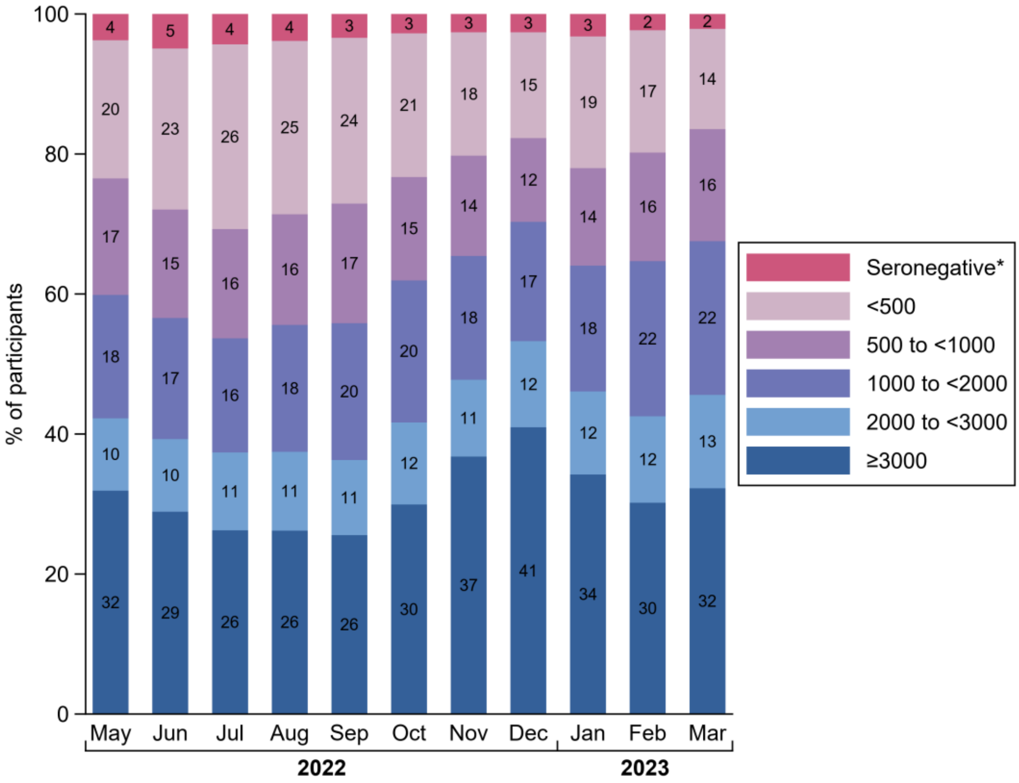

Figure S2. Percentage of participants in categories of Spike RBD IgG antibody titres in Binding Antibody Units per millilitre between May 2022 and March 2023. The analysis involved data of 37,065 measurements taken from 28,768 individuals. *Seronegativity corresponds to Spike RBD IgG antibody titres <7.1 Binding Antibody Units per millilitre (BAU/mL). All numbers in the legend are given in BAU/mL.
